# Ultrasound‐indicated and physical examination‐indicated cervical cerclage in twin versus singleton gestations

**DOI:** 10.1111/jog.16263

**Published:** 2025-03-11

**Authors:** Daphna Amitai Komem, Raanan Meyer, Itai Yagel, Daniel Shai, Roy Mashiach, Israel Hendler, Shali Mazaki‐Tovi, Yoav Yinon

**Affiliations:** ^1^ Department of Obstetrics and Gynecology, Sheba Medical Center, Tel‐Hashomer Affiliated to Sackler School of Medicine Tel Aviv University Tel Aviv Israel

**Keywords:** bulging membranes, cervical cerclage, preterm birth, short cervix, twin gestation

## Abstract

**Aim:**

To compare the safety and efficacy of ultrasound‐ and physical examination‐indicated cervical cerclage in twin versus singleton gestations.

**Methods:**

A retrospective cohort study of all ultrasound‐indicated (cervical length ≤ 25 mm) and physical examination‐indicated cerclage cases performed over a 9‐year period. The primary outcome was the time interval from cerclage placement to delivery.

**Results:**

The study cohort included 94 singleton and 16 twin pregnancies. The time interval from cerclage placement to delivery was comparable in singleton and twin gestations (14.77 vs. 12.07 weeks, *p* = 0.11), as were the rates of preterm births before 28 and 32 weeks. The rate of alive newborns >24 weeks was lower in the twin group (71.9% vs. 88.3%, *p* = 0.028). Regression analysis identified that cervical dilation, but not twin gestation, was the only factor independently associated with an increased risk for birth before 32 weeks.

**Conclusion:**

Ultrasound‐indicated and physical examination‐indicated cerclage had comparable efficacy in prolonging pregnancy in twin and singleton gestations, though live birth rates were lower in twins.

## INTRODUCTION

The prevalence of twin gestation has markedly increased over the last three decades, largely due to the expanded use of reproductive technology.^1^, ^2^ The rise in multiple gestations has been linked to increased risks of neonatal morbidity and mortality, predominantly due to preterm births (PTB). Moreover, half of twin pregnancies deliver before the 37th week of gestation, with 9% delivering before 32 weeks.^3^ Notably, a sonographic short cervix stands as a well‐established risk factor for PTB, irrespective of whether it is a singleton or a twin gestation.^4^ A cervical length of 20 mm or less at mid‐gestation in twin pregnancies is associated with a rate of 24% for PTB <32 weeks, increasing to 66% if the cervical length is 10 mm.^5^

The efficacy of various interventions aimed at treating or preventing PTB in twin gestations, ranging from bed rest and tocolytics to progesterone and cerclage, remains a subject of controversy.^6^, ^7^, ^8^, ^9^, ^10^, ^11^, ^12^, ^13^, ^14^ Cerclage placement following physical examination or ultrasound indication has been shown to increase the interval from cerclage placement to delivery in singleton gestations with imminent preterm delivery during the second trimester.^15^, ^16^, ^17^, ^18^ Nevertheless, the use of cerclage in twin pregnancies is controversial, and the existing literature offers sparse and inconclusive evidence regarding its efficacy in twin gestation. Previous studies have shown that cervical cerclage in twin pregnancies may be harmful and could be associated with an increased risk for PTB,^7^, ^19^ while a more recent systematic review supports its use, especially among women with cervical length <15 mm or dilated cervix >10 mm.^14^ However, the conclusions of this review were based exclusively on data from observational studies, whereas randomized clinical trials (RCT) have shown an increased risk of PTB and adverse perinatal outcomes.^20^ A recent RCT showed that physical examination‐indicated cerclage significantly reduced the risk of PTB and perinatal mortality in twin gestations.^13^ Given the inconsistency of the current data, we aimed to evaluate the safety and efficacy of ultrasound‐ and physical examination‐indicated cervical cerclage placement in twin versus singleton gestations.

## METHODS

**FIGURE 1:**
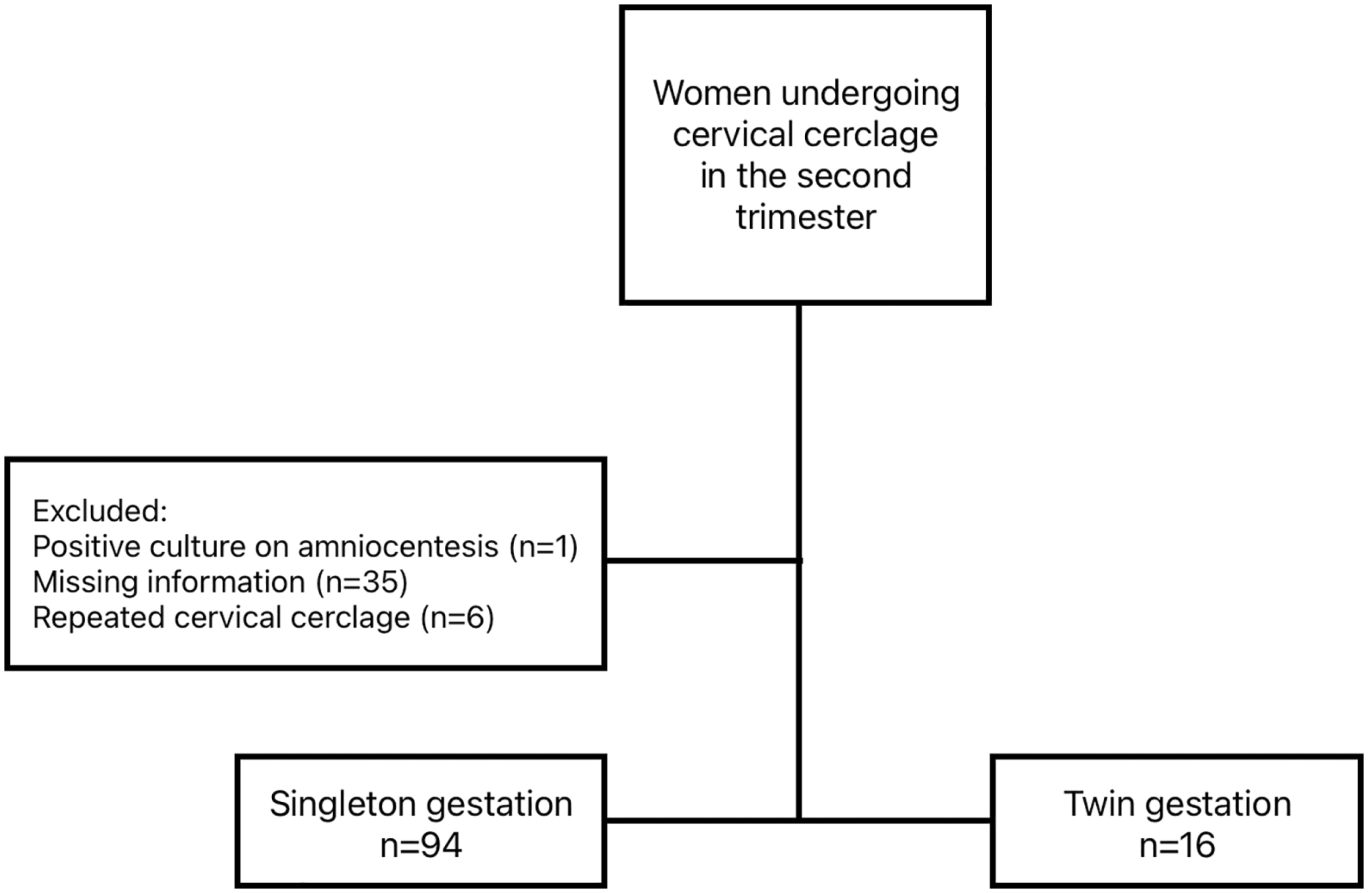
Patient flowchart.

A retrospective cohort study was conducted at a single tertiary referral center including all women who underwent either ultrasound‐indicated or physical examination‐indicated cerclage between 2011 and 2019. Ultrasound‐indicated cerclage was defined as cervical length ≤25 mm as indicated by previous studies.^4^, ^13^ Physical examination‐indicated cerclage was defined as cervical dilation ≥1 cm on manual exam. A total of 116 women were included and were divided into singleton (*n* = 94) and twin pregnancies (*n* = 16). The latter group included 13 dichorionic and three monochorionic twin pregnancies. Exclusion criteria included women who underwent history‐indicated cervical cerclage, cases with incomplete medical records (*n* = 35), patients who had a positive culture on amniocentesis before cerclage placement (*n* = 1), and women who underwent a second cerclage (*n* = 6). Figure 1 describes the patient flowchart. Pregnancy and neonatal outcomes were compared between the two groups.

### Data collection

The medical records of all women were reviewed, and data regarding patient history, cervical cerclage, and delivery were extracted. Pertinent demographic data included maternal age, prepregnancy body mass index, comorbidities, gravidity, parity, and mode of conception. Past obstetrical and gynecological risk factors for PTB, including a history of PTB, uterine anomalies, conization, hysteroscopy, chronic hypertension, and smoking were also recorded. According to our protocols, cervical length is routinely measured between 17 and 24 weeks, typically during the anatomical scan between 20 and 23 weeks. If cervical length is found to be shorter than 25 mm, the patient is referred for further evaluation.

Physical examination and ultrasound characteristics before cerclage placement included cervical length measured by transvaginal ultrasound, cervical dilation by digital examination, presence of bulging membranes on speculum exam, presence of sludge and funneling on ultrasound, and gestational age at the time of presentation for cerclage placement, as well as progesterone treatment. Amniocentesis was performed in select cases prior to cerclage placement to rule out intrauterine infection. According to our department's protocol, amniocentesis should be considered before emergency cerclage, particularly in the presence of bulging membranes, to rule out intrauterine infection.

The technique used for cervical cerclage was performed at the discretion of the operating physician and included either Shirodkar or McDonald techniques.^21^, ^22^ As part of our department's protocol, patients received perioperative prophylactic antibiotic treatment (1 g Cefazolin intravenously). Pregnancy and delivery outcomes collected included the occurrence of preterm premature rupture of membranes (PPROM) or chorioamnionitis, termination of pregnancy due to PPROM prior to 24 weeks of gestation or due to chorioamnionitis, time interval from cerclage to delivery, gestational age at delivery, and mode of delivery.

Neonatal data included live birth after 24 weeks, gender, birthweight and birthweight percentiles, 1‐ and 5‐min Apgar scores, umbilical cord pH, mechanical ventilation use, neonatal intensive care unit admission, duration of hospitalization, and neonatal mortality within the first 24 h and the first month of life.

The primary outcome was defined as the time interval between cervical cerclage placement and the time of delivery. Secondary outcomes were gestational age at the time of delivery, the rate of PTB prior to the 24th, 28th, 32nd, and 37th weeks of gestation, alive newborn deliveries after 24 weeks, and a composite adverse neonatal outcome defined as one or more of the following: neonatal intensive care unit admission, mechanical ventilation, and neonatal mortality. The time interval from cerclage to delivery by cerclage indication was compared between and within groups. The study protocol was approved by the local Institutional Review Board (5587‐18). Informed consent was deemed not required due to the retrospective nature of the study.

### Statistical analysis

Categorical variables were reported as frequency and percentage. Continuous variables were evaluated for normal distribution using histogram and Q‐Q plot and reported as mean and standard deviation (normally distributed variables) or median and interquartile range (skewed variables). Chi‐square test and Fisher's exact test were used to compare categorical variables between the two groups. Independent samples *t*‐test and Mann–Whitney test were used to compare continuous variables between the groups. Multivariate logistic regression was applied to study the association between type of pregnancy (twins vs. singleton), gestational age at the time of cerclage placement, cervical dilatation, and presence of bulging membranes and delivery before 28, 32, and 37 weeks of gestation. Adjustment was conducted for maternal age, pre‐gestational body mass index, and parity. All statistical tests were two sided, and *p* < 0.05 was considered statistically significant. SPSS software (IBM SPSS Statistics for Windows, version 23, IBM corp., Armonk, NY, USA, 2015) was used for all statistical analyses.

## RESULTS

Demographic and pregnancy characteristics are shown in Table 1a. Maternal age, body mass index, nulliparity, history of PTB, as well as medical risk factors for PTB were comparable between the two groups. Within the twins group, most women conceived through in vitro fertilization, whereas in the singleton group, most women conceived spontaneously.

**TABLE 1:** Demographic and clinical characteristics of twins and singleton pregnancies.

	Singletons (*n* = 94)	Twins (*n* = 16)	*p* value
(a) Patient characteristics			
Age, years	34.1 ± 6.2	31.2 ± 5.8	0.082
Nulliparous	53 (56.4%)	13 (81.2%)	0.096
Pre‐pregnancy BMI, kg/m^2^	22.2 [19.8, 26.6]	22.0 [20.8, 24.4]	0.897
Hx of PTB	20 (21.3%)	2 (12.5%)	0.52
Medical risk factors for PTB^a^	73 (77.7%)	13 (81.2%)	1.00
Mode of conception			<0.001
Spontaneous	57 (60.6%)	2 (12.5%)	
Ovulation induction	4 (4.3%)	2 (12.5%)	
IVF	22 (23.4%)	11 (68.8%)	
IVF ED	9 (9.6%)	0 (0.0%)	
IUI	2 (2.1%)	1 (6.2%)	
GDM			0.482
GDMA1	15 (16.0%)	1 (6.2%)	
GDMA2	6 (6.4%)	0 (0.0%)	
Hypertensive disorders of pregnancy^b^	2 (2.1%)	0 (0.0%)	1
Intrahepatic cholestasis of pregnancy	1 (1.1%)	0 (0.0%)	1
(b) Cerclage characteristics			
GA at cerclage placement, weeks	20.6 [18.6, 22.5]	20.9 [18.8, 22.3]	0.926
Presence of cervical dilation	36 (38.3)	8 (50.0)	0.416
Cervical length, mm	14.0 (0–25)	11.5 (1–24)	0.822
Bulging membranes	30 (31.9%)	7 (43.8%)	0.397
Indication for cerclage			
Short cervical length	58 (61.7%)	8 (50.0%)	0.416
Dilation and/ or bulging membrane	36 (38.3%)	8 (50.0%)	
Sludge	16 (17.0%)	2 (12.5%)	1.00
Funneling	49 (52.1%)	11 (68.8%)	0.281
Cerclage type			
McDonald	18 (19.1%)	3 (18.8%)	1.00
Shirodkar	76 (80.9%)	13 (81.2%)	
Progesterone treatment	62 (66.0%)	12 (75.0%)	0.574
Amniocentesis before cerclage	17 (18.08%)	0 (0.0%)	0.126
(c) Perinatal outcome			
Time interval from cerclage to delivery, weeks	16.4 [12.0, 18.7]	14.1 [7.5, 16.1]	0.085
GA at delivery, weeks	37.9 [33.4, 39.0]	36.1 [28.6, 37.2]	0.014
<24 weeks	10 (10.6%)	4 (25.0%)	0.12
<28 weeks	18 (19.1%)	4 (25.0%)	0.735
<32 weeks	21 (22.3%)	5 (31.2%)	0.525
<37 weeks	39 (41.5%)	10 (62.5%)	0.173
Mode of delivery			
Vaginal	73 (77.7%)	11 (68.8%)	0.525
Cesarean	21 (22.3%)	5 (31.2%)	
PPROM	14 (14.9%)	3 (18.8%)	0.711
Chorioamnionitis	12 (12.7%)	4 (25.0%x)	0.246

*Note*: Values are presented as *n* (%), mean ± SD, median [IQR], or median (min–max).

Abbreviations: BMI, body mass index; ED, egg donation; GA, gestational age; GDM, gestational diabetes mellitus; IUI, intrauterine insemination; IVF, in vitro fertilization; NICU, neonatal intensive care unit; PPROM, preterm premature rupture of membranes; PTB, preterm birth.

^a^ Positive history of any of the following: D&E—Dilation and evacuation, cervical preparation, conization, hysteroscopy, uterine anomaly, chronic hypertension, smoking.

^b^ Pregnancy induced hypertension, preeclampsia or HELLP syndrome.

Regarding cervical cerclage, the rate of indications (short cervix indicated by transvaginal ultrasound vs. cervical dilation by physical examination) as well as the gestational age at cerclage placement, did not differ significantly between the two groups (Table 1b). Similarly, the mean cervical length measured by transvaginal ultrasound was similar between the two groups; the presence of cervical dilatation, bulging membranes, sludge, and funneling was also comparable between the two groups. The majority of patients underwent the Shirodkar cerclage technique. The use of intravaginal progesterone application (either *Utrogestan* 200 mg twice daily or *Crinone* 8%, 1 application daily) did not differ between groups.

Delivery outcomes are presented in Table 1c. The time interval from cerclage placement to delivery did not differ between twins and singleton gestations (14.1 weeks [IQR 7.5, 16.1] vs. 16.4 weeks [IQR 12.0, 18.7], respectively, *p* = 0.085). As expected, women with twin gestations delivered earlier compared to women with singletons (37.9 weeks [IQR 33.4, 39.0] vs. 36.1 weeks [IQR 28.6, 37.2], *p* = 0.014), but the rates of PTB prior to 24, 28, 32, and 37 weeks did not differ between the two groups. Similarly, there was no difference in the rate of PPROM and chorioamnionitis. The rate of live births after 24 weeks was significantly lower in twin pregnancies (88.3% in twins vs. 71.9% in singltons, *p* = 0.028). As expected, the median birth weight was significantly lower in the twin group compared to the singleton group (2310.0 g [IQR 1322.0, 2670.0] vs. 2880.0 g [IQR 2355.0, 3320.0], *p* < 0.001). Umbilical cord pH, length of hospitalization, and the rate of neonatal intensive care unit admission, as well as mechanical ventilation use, neonatal mortality, and the rate of the composite adverse neonatal outcome were similar between the two groups (Table 2).

**TABLE 2:** Neonatal outcomes in singletons versus twins.

	Singleton (*n* = 94)	Twins (*n* = 32)	*p* value
Male gender	42 (48.8%)	16 (57.1%)	0.517
Birth weight, g	2880.0 [2355.0, 3320.0]	2310.0 [1322.0, 2670.0]	<0.001
Alive newborn deliveries ≥24 weeks	83 (88.3%)	23 (71.9%)	0.028
Apgar <7 at 5 min	7 (7.9%)	4 (14.3%)	0.292
Venous cord pH	7.3 ± 0.05	7.3 ± 0.04	0.071
NICU	8 (9.4%)	2 (6.7%)	1.00
Mechanical ventilation	7 (8.5%)	5 (16.7%)	0.299
Death during 1st month of life	1 (1.2%)	0 (0.0%)	1.00
Days in hospital	4.0 [3.2, 6.0]	5.0 [3.0, 11.0]	0.955
Composite neonatal complications^a^	18 (19.1%)	7 (21.9%)	0.799

*Note*: Values are presented as *n* (%), mean ± SD, median [IQR], or median (min–max).

Abbreviation: NICU, neonatal intensive care unit.

^a^ Composite adverse neonatal outcome included one or more of the following: NICU admission, mechanical ventilation, neonatal mortality.

A comparison between the time interval from cerclage to delivery by indications for cerclage between and within groups was performed (Table 3). Results show that among singleton and twin gestations, the time interval was significantly prolonged in cases of cervical shortening versus cervical dilation, and there was no significant difference between the two groups when compared by cerclage indications.

**TABLE 3:** Comparison of time interval from cerclage to delivery (a) within each group and (b) between twins and singletons by cerclage indication.

(a) Within each group						
	Twins			Singletons		
	Physical examination‐indicated (*n* = 8)	Ultrasound‐indicated (*n* = 8)	*p* value	Physical examination‐indicated (*n* = 36)	Ultrasound‐indicated (*n* = 58)	*p* value
Time interval from cerclage to delivery, weeks	8.6 ± 7.1	15.5 ± 4.7	0.040	12.3 ± 7.1	16.3 ± 5.0	0.005

(b) Between twins and singletons						
	Physical examination‐indicated			Ultrasound‐indicated		
	Twins (*n* = 8)	Singletons (*n* = 36)	*p* value	Twins (*n* = 8)	Singletons (*n* = 58)	*p* value
Time interval from cerclage to delivery, weeks	8.0 ± 6.4	12.3 ± 7.1	0.210	15.5 ± 4.7	16.3 ± 5.0	0.682

*Note*: Values are presented as mean ± SD.

Multivariate regression analysis (Table 4) revealed that cervical dilation at the time of cerclage placement was the only factor independently associated with an increased risk for PTB < 32 weeks of gestation (odds ratio [OR], 5.32; 95% confidence interval 1.35–20.94), while twin gestation was not associated with an increased risk for PTB < 32 weeks (OR, 1.36; 95% confidence interval 0.38–4.85).

**TABLE 4:** Multivariate analysis of factors associated with preterm birth <32 weeks.

Variable	OR (95% CI)	*p* value
Twin gestation	1.683 (0.490–5.779)	0.40
Gestational age at time of cerclage placement	0.882 (0.741–1.050)	0.10
Cervical dilation	3.630 (1.025–12.855)	0.04
Bulging membranes	2.308 (0.714–7.455)	0.10

*Note*: Adjustment was made for maternal age, pre‐gestational body mass index, and parity.

## DISCUSSION

This study examined the impact of ultrasound‐ and physical examination‐indicated cerclage on obstetric and neonatal outcomes in 16 twin versus 94 singleton gestations. Our findings indicate that the median interval from cerclage placement to delivery did not significantly differ between groups. Nevertheless, the rate of live newborns after 24 weeks of gestation was notably lower in the twins group compared to the singleton group. As anticipated, women with twin pregnancies delivered earlier than those with singleton pregnancies; however, the rate of delivery prior to 24, 28, 32, and 37 weeks of gestation, as well as the composite adverse neonatal outcomes, were comparable between the two groups.

Given the inconclusive nature of current literature on the efficacy of midtrimester cerclage in twin pregnancies, the decision to place a cervical cerclage in these cases is often complex and based on multiple factors as well as clinical judgment and the physician's expert opinion. Our results are consistent with previous studies indicating a beneficial effect of cervical cerclage when comparing twin to singleton gestations.^13^, ^23^, ^24^, ^25^, ^26^ These studies demonstrated favorable obstetric outcomes for physical exam‐indicated cerclage in twin gestations vs. singletons and reported a shorter median time from cerclage placement to delivery than our study (Miller et al.: 64 vs. 83 days, Rebarber et al.: 92 vs. 106 days, our study: 99 vs. 115 days).^23^, ^25^ This difference might be attributed to minor variations in management, cerclage technique, and our study population, which included both physical examination‐ and ultrasound‐indicated cervical cerclage. Furthermore, a recent meta‐analysis showed that the use of cerclage in twin pregnancies is beneficial for pregnancy prolongation and the reduction of the risk of PTB in cases with a cervical length of <15 mm or a dilated cervix. According to this meta‐analysis, previous RCTs showed a beneficial effect of ultrasound‐indicated cerclage in twin pregnancies, while cohort studies showed contradictory effects. This discrepancy precludes a definite conclusion regarding the management of this subgroup.^14^ Our results are consistent with these studies, showing improvement in the cerclage to delivery interval and neonatal outcomes in cases of sonographic short cervix as well as cervical dilation and stand in contrast with earlier studies, in which cerclage placement in twin pregnancies was associated with a significant two‐fold increase in the rate of PTB as compared to no cerclage.^7^

Unlike previous studies that typically focused on a single indication,^23^ our cohort included both physical examination‐ and ultrasound‐ indicated cerclage, potentially providing a more comprehensive representation of the clinical scenario. When stratifying by indication, the time interval from cerclage to delivery was longer for women with a short cervix than for those with cervical dilation. Barbosa et al. investigated the efficacy of cerclage in physical examination‐indicated versus ultrasound‐indicated cerclage in twin pregnancies and revealed consistent results, showing an extended latency period particularly in cases indicating a short cervical length.^27^

Furthermore, our study highlights cervical dilation as the sole significant independent risk factor for PTB < 32 weeks. These findings align with Han et al. that compared perinatal outcomes between cerclage and no cerclage in twins, revealing cervical dilation as the primary significant risk factor for PTB < 32 weeks.^28^ Our study does have some limitations. The retrospective design of this study carries inherent biases, and the relatively modest sample size of our study may explain some of the absence of statistically significant differences between the two groups secondary to lack of statistical power. The difference in gestational age at delivery between singletons and twins may represent the inherent risk of earlier delivery in twin pregnancies. However, we cannot rule out the possibility that cervical cerclage may be less effective in twins compared to singletons. While this study acknowledges its limitations and potential constraints on generalizability, the congruence with previous studies suggests that our findings might extend beyond the confines of our institution. The strength of this study lies in the detailed medical record enabling comprehensive data extraction and its nature as a single‐center study with consistent antenatal and neonatal management protocols.

Our findings show a comparable time interval from cerclage placement to delivery in both twins and singletons; however, gestational age at delivery and live birth rates were lower in twins. This suggests that ultrasound‐ and physical examination‐indicated cervical cerclage may prolong pregnancy in women with twin gestations similar to singleton gestations. However, the lower rate of live newborns underscores the importance of counseling patients accordingly. Randomized prospective studies are urgently needed to establish the efficacy of cerclage placement in twin pregnancies with a short and/or dilated cervix.

## AUTHOR CONTRIBUTIONS

**Daphna Amitai Komem:** Conceptualization; data curation; investigation; methodology; project administration; validation; visualization; writing – original draft; writing – review and editing. **Raanan Meyer:** Conceptualization; formal analysis; investigation; methodology; writing – review and editing. **Itai Yagel:** Data curation; investigation; writing – review and editing. **Daniel Shai:** Data curation; investigation; writing – review and editing. **Roy Mashiach:** Data curation; investigation; methodology; writing – review and editing. **Israel Hendler:** Methodology; project administration; writing – review and editing. **Shali Mazaki‐Tovi:** Conceptualization; investigation; methodology; project administration; writing – review and editing. **Yoav Yinon:** Conceptualization; formal analysis; investigation; methodology; project administration; supervision; validation; writing – review and editing.

## CONFLICT OF INTEREST STATEMENT

The authors have no conflicts of interest to disclose.

## Data Availability

The data that support the findings of this study are subject to restrictions imposed by the ethics committee of Sheba Medical Center, prohibiting public access due to privacy concerns and ethical guidelines. However, data can be made available for researchers who meet the necessary criteria for access to confidential data, in accordance with these restrictions. Interested researchers can request access by contacting the cooresponding author.
